# COVID-19 Infection Alters the Microbiome: Elite Athletes and Sedentary Patients Have Similar Bacterial Flora

**DOI:** 10.3390/genes12101577

**Published:** 2021-10-04

**Authors:** Gergely Babszky, Ferenc Torma, Dora Aczel, Peter Bakonyi, Zoltan Gombos, Janos Feher, Dóra Szabó, Balázs Ligeti, Sándor Pongor, Laszlo Balogh, Anikó Pósa, Zsolt Radak

**Affiliations:** 1 Research Institute of Sport Science, University of Physical Education, 1123 Budapest, Hungary; babszky.gergely@tf.hu (G.B.); torma.ferenc@tf.hu (F.T.); aczel.dora555@gmail.com (D.A.); bakonyi.peti@gmail.com (P.B.); gzoltan5@gmail.com (Z.G.); 2 Department of Ophthalmology, Sapienza University of Rome, 00185 Rome, Italy; j.feher@libero.it; 3 Institute of Medical Microbiology, Semmelweis University, 1123 Budapest, Hungary; szabo.dora@med.semmelweis-univ.hu; 4 Faculty of Information Technology and Bionics, Pázmány Péter Catholic University, 1123 Budapest, Hungary; obalasz@gmail.com (B.L.); pongor@icgeb.org (S.P.); 5 Institute of Sport Science, University of Debrecen, 4000 Debrecen, Hungary; balogh.laszlo@sport.unideb.hu; 6 Interdisciplinary Excellence Center, Department of Physiology, Anatomy and Neuroscience, Faculty of Science and Informatics, University of Szeged, 6700 Szeged, Hungary; paniko@bio.u-szeged.hu; 7 Faculty of Sport Sciences, Waseda University, Tokorozawa 2-579-15, Japan

**Keywords:** *Bacteroidetes*, COVID-19, microbiome, exercise, inflammation

## Abstract

Regular exercise can upgrade the efficiency of the immune system and beneficially alter the composition of the gastro-intestinal microbiome. We tested the hypothesis that active athletes have a more diverse microbiome than sedentary subjects, which could provide better protection against COVID-19 during infection. Twenty active competing athletes (CA) (16 male and 4 females of the national first and second leagues), aged 24.15 ± 4.7 years, and 20 sedentary subjects (SED) (15 male and 5 females), aged 27.75 ± 7.5 years, who had been diagnosed as positive for COVID-19 by a PCR test, served as subjects for the study. Fecal samples collected five to eight days after diagnosis and three weeks after a negative COVID-19 PCR test were used for microbiome analysis. Except for two individuals, all subjects reported very mild and/or mild symptoms of COVID-19 and stayed at home under quarantine. Significant differences were not found in the bacterial flora of trained and untrained subjects. On the other hand, during COVID-19 infection, at the phylum level, the relative abundance of *Bacteroidetes* was elevated during COVID-19 compared to the level measured three weeks after a negative PCR test (*p* < 0.05) when all subjects were included in the statistical analysis. Since it is known that *Bacteroidetes* can suppress toll-like receptor 4 and ACE2-dependent signaling, thus enhancing resistance against pro-inflammatory cytokines, it is suggested that *Bacteroidetes* provide protection against severe COVID-19 infection. There is no difference in the microbiome bacterial flora of trained and untrained subjects during and after a mild level of COVID-19 infection.

## 1. Introduction

Coronaviruses are one of the largest (27–34 kilobases) positive-stranded non-segmented RNA viruses, named after the ~120 nm diameter envelope (resembles solar corona) around the nucleic acid–protein complex. They have been known since the 1930s. The ongoing pandemic caused by a new strain of severe acute respiratory syndrome coronavirus 2 (SARS-CoV-2) strikingly increased the interest in this virus family. The strain isolated in 2019—named coronavirus disease 2019 (COVID-19) by the World Health Organization (WHO)—causes upper and lower respiratory tract disease. Because virus infections have been reported worldwide, the WHO has declared a global health emergency [1]. SARS-CoV-2 has ~30 pb single-stranded RNA genomes [2]. After entry into the host cell, RNA binds to the host cell ribosome for translation. Two-thirds of the genomes encode a large pre-protein, which is cleaved to generate 16 non-structural proteins. The last third of the genomes encode the so-called spike, envelope, matrix, nucleocapsid and non-structural proteins [1,2,3]. It is believed that the entry of SARS-CoV-2 into human tissues is facilitated via angiotensin-converting enzyme 2 (ACE-2), which is highly expressed in the intestine, especially in colonocytes of healthy subjects and in patients with inflammatory bowel disease [4,5]. Recognition of viral RNA fragments by the host immune system plays a key role in limiting virus replication and spreading as well as in the outcome of infection-induced diseases. Transmission of the virus has been rapid, especially for the new lineage of the SARS-CoV-2 virus (named B.1.1.7, first sequenced in the southeast of England, and called the British variant) that became the dominant variant in Europe. The clinical manifestations of COVID-19 range from asymptomatic, mild to moderate systematic symptoms and severe abnormalities observed in immunodeficient and older patients [6,7]. By checking the profile of those with the severest symptoms it becomes apparent that obesity, diabetes or physical inactivity all significantly worsen the condition of patients [8].

Although a higher level of physical fitness or regular physical activity cannot prevent the spread of COVID-19, the results of some reports suggest that regular exercise decreases the rate of mortality of patients suffering from COVID-19 [9] and might suppress the severity of this infection [10,11]. The general hypothesis is that regular exercise decreases the rate of obesity, improves insulin sensitivity and stress tolerance, and enhances the capability of the immune system [12,13,14,15]. Regular exercise impacts the immune system via complex regulations, which involve the proper adjustment of pro- and anti-inflammatory cytokine and neopterin production [16]. It also has been shown that exercise-induced modulation of metabolism alters proinflammatory responses in macrophages and the modulation can involve the energy sensor 5′-adenosine-monophosphate-activated protein kinase (AMPK) [15]. Sirtuin 1 (SIRT1), the activity and contents of which readily respond to exercise training [17], promotes anti-inflammatory and tolerance programs in multiple immune cell types [18]. Metabolites, which are the products of catabolic processes of proteins, carbohydrates and fats are involved in immune defense as well as acute-phase responses and complement the activation of humoral responses mediated by circulating immunoglobulins [15].

The gastro-intestinal microbiome is a powerful part of the human immune system; indeed, the microbiome is implicated in COVID-19 [19,20,21,22]. It has been reported that gut microbiome composition of discharged COVID-19 patients differs from that of the general population at both the phylum and genera levels, characterized by a lower proportion of Firmicutes and Actinobacteria and a higher proportion of *Bacteroidetes* and Proteobacteria [23].

Both acute and regular exercise impact the gastro-intestinal microbiome [24,25,26,27,28]. However, the possible link between the severity of COVID-19 and exercise-altered microbiome is not known. Therefore, we have collected fecal samples of competing athletes and age-matched sedentary subjects during and three weeks after COVID-19 infection in order to investigate whether exercise-induced alterations of the gastro-intestinal microbiome are associated with different responses to COVID-19 infection.

## 2. Materials and Methods

The present study was approved by the local ethics committee (TE-KEB/30/2020) and done according to the Declaration of Helsinki. Subjects were voluntarily recruited for the study. The subjects read and signed the protocol for this investigation.

Twenty active competing athletes (CA) (16 male and 4 females of the national first and second leagues), aged 24.15 ± 4.7 years, and 20 sedentary subjects (SED) (15 male and 5 females), aged 27.75 ± 7.5 had been diagnosed as positive for COVID-19 by a PCR test. At the time of the present investigation, a significant part of the infection in Hungary was caused by the B.1.1.7 variant of theCOVID-19 virus, first sequenced in the southeast of England, and called the α variant.

Subjects from both groups were asked to provide information concerning their exercise and nutritional habits and the severity of symptoms of COVID-19 that they experienced. The average training time per week was 14.4 ± 6.2 h in CA and 1.25 ± 0.9 h in the SED group. All subjects filled out a questionnaire on nutritional habits.

All of the infected subjects stayed at home under quarantine and evaluated the severity of their infection using the scale suggested by the National Institutes of Health (USA), in short: 1, asymptomatic infection, no symptoms that are consistent with COVID-19; 2, mild illness, various signs and symptoms; 3, moderate illness, evidence of lower respiratory disease during clinical assessment; 4, severe illness, with SpO_2_ < 94% in room air at sea level and high fever; and 5, critical illness, requiring medical treatment to maintain organ function and life (Table 1).

Subjects not under any medication with diarrhea, auto-immune diseases, asthma, cardiovascular diseases, metabolic diseases and neurodegenerative diseases were excluded from the study.

Fecal samples were collected five to eight days following diagnosis of COVID-19 by the PCR test and three weeks after a negative COVID-19 PCR-test. Subjects collected the samples whilst following instructions at home, they were picked up within one hour after collection and then stored at -80 C degrees until analysis. Stool samples of 100 mg were used for DNA extraction. The collected samples were stored at -80 C degrees until analysis.

### 2.1. Library Preparation and Identification of Prokaryotic Species

The DNA from stool samples was isolated by a QIAmp Fast DNA Stool Mini Kit (Qiagen, Beverly, MA, USA). Fragment libraries were constructed from purified DNA using NEBNext Fast DNA Fragmentation & Library Prep Set for Ion Torrent (New England Biolabs) according to the manufacturer’s instructions. Briefly, DNA was enzymatically digested and the fragments were end-repaired. Ion Xpress Barcode Adaptors (Life Technologies, Carlsbad, CA, USA) were than ligated and the template fragments were size-selected using Agincourt AMPure XP magnetic beads (Beckman Coulter, Pasadena, CA, USA). Adaptor-ligated fragments were then PCR amplified, cleaned up using AMPure beads, quality checked on D1000 ScreenTape and reagents using a TapeStation instrument (Agilent, Santa Clara, CA, USA) and finally quantitated using the Ion Library TaqMan Quantitation Kit (Life Technologies). The library templates were prepared for sequencing using the Ion OneTouch protocols and reagents from Life Technologies. Briefly, library fragments were clonally amplified onto Ion Sphere Particles (ISPs) through emulsion PCR and then enriched for template-positive ISPs. More specifically, PGM emulsion PCR reactions utilized the Ion PGM Hi-Q OT2 Kit (Life Technologies), and as specified in the accompanying protocol, emulsions and amplification were generated using the Ion OneTouch System (Life Technologies). Enrichment was completed by selectively binding the ISPs containing amplified library fragments to streptavidin-coated magnetic beads, removing empty ISPs through washing steps and denaturing the library strands to allow for the collection of template-positive ISPs. For all reactions, these steps were accomplished using the Life Technologies ES module of the Ion OneTouch System. Template-positive ISPs were deposited onto Ion 318 chips (Life Technologies); finally, sequencing was performed with the Ion PGM Hi-Q view OT2 Kit (Life Technologies).

### 2.2. Bioinformatics Analysis

Essentially, the taxonomic classification and sequence data preprocessing was carried out by the HuGe-F laboratory, using the RAPtorU v3.0 pipeline. Briefly, it applied QIIME 1 [29] for demultiplexing, TagCleaner v.06 [30] for primer trimming and DADA2 [31] for denoising the reads. The analyses yielded 4.9125 GB above the quality threshold (Phred score > 30), 52,754 reads/sample on average and 5331 distinct ASVs (amplicon sequence variant covering 659 taxa). The ASVs were assigned to taxonomy using the RDP [32] algorithm with default parameters.

### 2.3. Statistics

The rare (maximum of 10% were present in all samples) and low-abundance (support of less than 100 reads) taxa were discarded from the subsequent analysis as well as the samples with low coverage (#reads/sample < 30,000). After the filtering process a Bayesian-multiplicative replacement of zeros was carried out using the zCompositions R package, which was followed by a centered log-ratio (CLR) transformation of count and ratio values as implemented in scikit-bio.

The visualization of the microbiome was carried out with biplot PCA (principal component analysis) using the CLR values as an input matrix (scikit-learn v0.24) [33].

The Shannon index was used to measure α diversities, which quantify the entropy of the distributions of taxa proportions.

The compositional similarities between the different groups were investigated with PERMANOVA and the differential abundance testing was done using the Wilcoxon signed-rank test (comparing biome before–after COVID) and the Wilcoxon rank-sum test.

## 3. Results

Two subjects with low levels of reads were excluded from the study. The severity of COVID-19 ranged between grade 2, (mild infection) and grade 4 (severe illness). Interestingly enough, the only two subjects with severe illness symptoms of COVID-19 were professional athletes (Table 1).

**Figure 1:**
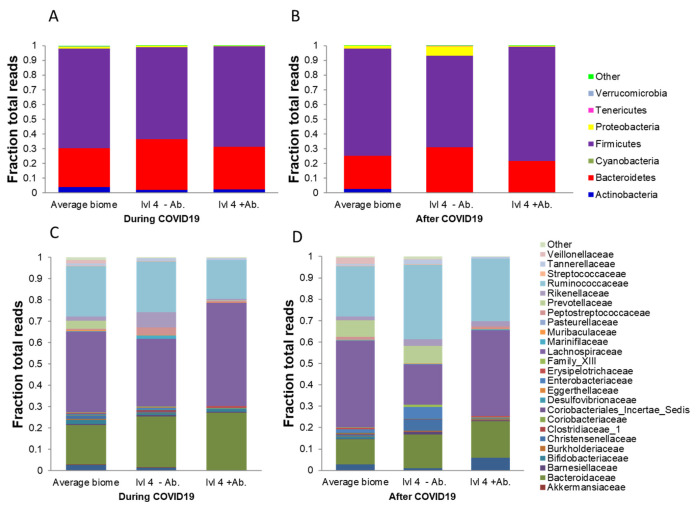
The microbiome levels of trained and untrained individuals during and after COVID-19 infection. The first bar shows the microbiome flora at the phylum level of the (**A**,**B**) panels and at the family level of the (**C**,**D**) panels of all of the subjects (*n* = 38). The second bar shows the values of the subject who was in serious condition (with level 4: severe illness, with SpO_2_ < 94% in room air at sea level and high fever) without antibiotic treatment (lvl4 − Ab), while the third bar represents the values of the other subject with level 4 infection but with antibiotic treatment (lvl4 + Ab).

The present investigation only focused on COVID-19 subjects who were assigned to home quarantine. Figure 1 shows the bacterium contents at the phylum level. The first bar at panel A and panel B shows the average distribution of the members of phylum during and after COVID-19 infection. The second bar show the members of phylum of subjects who had with severe illness symptoms without antibiotic treatment, while the third bar represents the same for the other subjects with severe illness symptoms but with antibiotic treatment. The C and D panels of Figure 1 display the bacterial members at the family level. The microbiome analysis at the phylum level revealed that although the bacterial flora of the same subjects during and after COVID-19 infection was quite similar, assessed by correlation (Figure 1), even antibiotic treatment did not show significant alterations.

**Figure 2:**
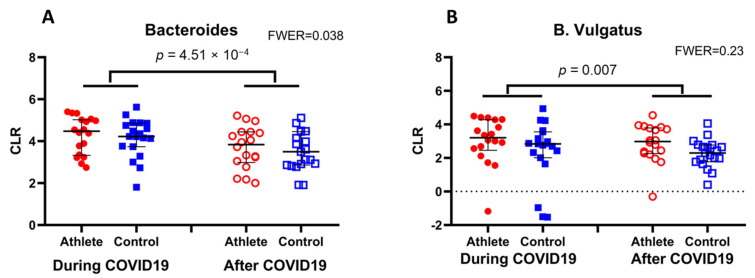
The *Bacteroidetes* levels were higher during COVID-19 infection than after recovery. The only significant difference was observed at the phylum (**A** panel) and species (panel **B**) level: the *Bacteroidetes* had higher abundance at phylum and *B. Vulgatus* at species level during COVID-19 infection than two–three weeks after a negative PCR test (*p* < 0.05). *n* = 38. The confidence at *Bacteroidetes* case was 99.19%. CLR: centered log-ratio, *n* = 38.

At the phylum level the relative concentration of *Bacteroidetes* was elevated during COVID-19 infection compared to levels measured three weeks following a negative PCR test (*p* < 0.05) when all subjects were included in the statistical analysis (Figure 2).

**Figure 3:**
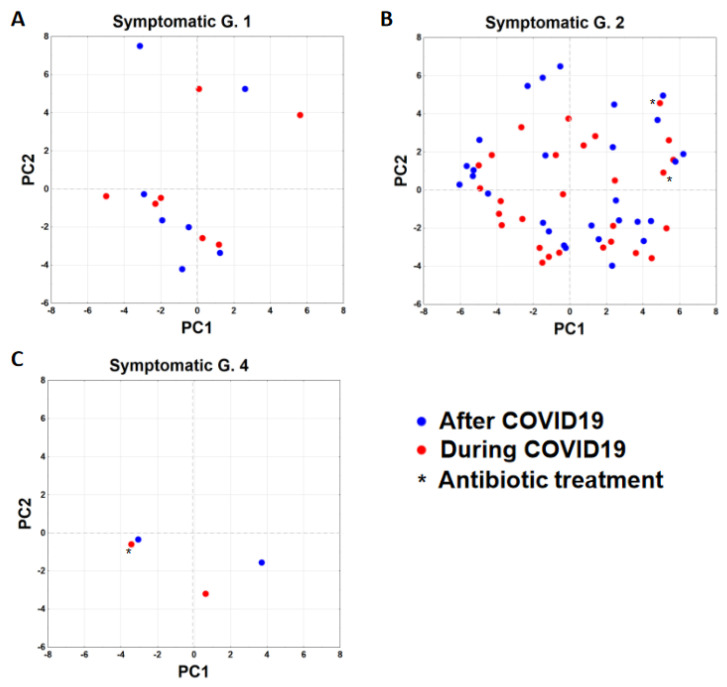
Principal component analysis of gut microbiota composition of subjects. Principal component analysis of the bacterial composition data. Subjects are divided into symptomatic group 1 (panel **A**), 2 (panel **B**) and 4 (panel **C**) and plotted according to the two principle component with the highest explanation of the variance (PC1: 14.5% and PC2: 10.5% respectively). Red dots representing samples taken During COVID19 infection, blue dots showing samples taken after infection. Asterisk marks individuals who was treated with antibiotics.

At the species level, *B. vulgatus* was elevated during infection when compared to samples gained after recovery from disease (Figure 2). Principal component analysis of gut microbiota composition of subjects with PCR confirmed COVID-19 during and after recovering from disease. The data points do not show an apparent pattern of group-specific clustering (Figure 3).

**Figure 4:**
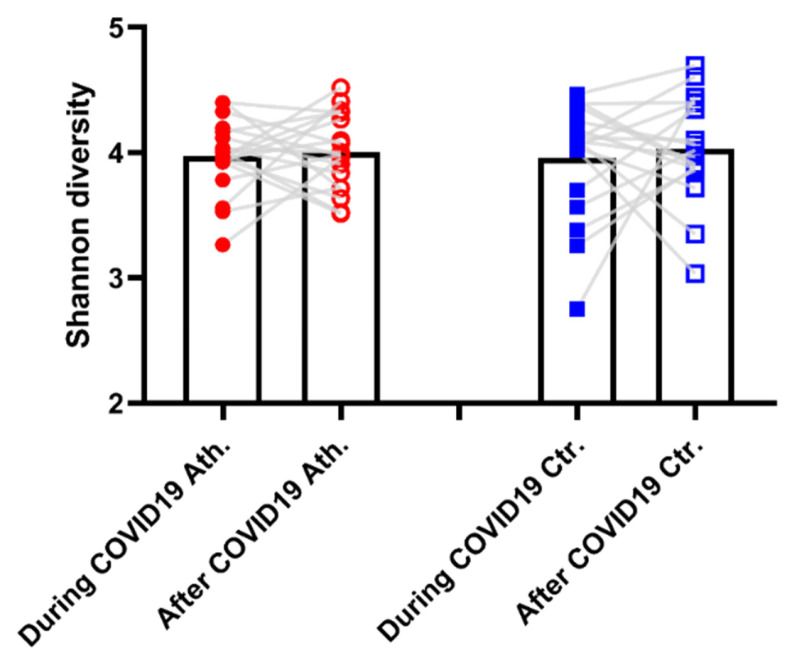
The Shannon diversity of the samples. The Shannon diversity was evaluated and the results are shown on the figure. The Shannon diversity of the microbiome was independent from training status or COVID-19 infection. *n* = 38.

Significant differences were not found in the bacterial flora of trained and sedentary groups, and we could not identify any bacteria which could be linked to the severity of COVID-19 infection. The Shannon diversity of the microbiome was independent from training status or COVID-19 infection (Figure 4).

Two subjects reported severe symptoms, and their microbiome flora and the average flora are presented in Figure 1.

Based on the provided data on nutritional habits, significant alterations were not present at the examined periods, during and after COVID-19 infection. The average daily consumption of carbohydrate, protein and fat during the COVID-19 infection of the athletes was 158.6 g, 74.5 g and 75.6 g, and after a negative PCR test was 158.7 g, 65.5 g and 75 g, respectively. The daily carbohydrate, protein and fat consumption of sedentary subjects during COVID-19 infection was 144.2 g, 52.5 g and 56.7 g, and was 146.2 g, 53.2 g and 56.9 g after infection, respectively.

## 4. Discussion

It is known that the microbiome plays an important role in the immune system, and it is suggested that regular exercise has the capacity to boost the immune system [10,15,34]. On the other hand, our data revealed no significant difference in the microbiome of competing athletes and sedentary subjects during and after COVID-19 infection. Since the microbiome-related immune response is just one part of the immune system, this finding does not mean that regular exercise-related adaptive responses would not include increased immunosurveillance.

It is clear that regular physical training and high levels of fitness do not protect against COVID-19 infection, and due to the nature of sport, frequent physical contact and team preparation, the rate of COVID-19 transmission is relatively high among team athletes [35]. However, it is suggested that the combination of high fitness levels and frequent sauna baths is associated with a substantially lowered future pneumonia risk compared to each modality alone, which can lower the severity of COVID-19 [36]. In general, the combination of young age and involvement in elite sport appears to result in asymptomatic COVID-19 infection [37]. Interestingly, in our study two subjects reported severe symptoms and both were elite water polo players, one of which received antibiotic treatment in the hospital. Since antibiotic treatment can readily alter the microbiome, these data were separately displayed in Figure 1. Probably because the antibiotic treatments were short, we could not find a very notable difference in the bacterial flora of the treated subject.

Although we could not detect differences in the microbiome content of trained and sedentary individuals during or after COVID-19 infection, the microbiome during infection was somewhat different than three weeks following infection. At the phylum level, the relative concentration of *Bacteroidetes* was elevated during COVID-19 infection. Earlier it was suggested that gut microbial communities possess a robust toll-like receptor 4 (TLR4) signaling capacity against agents which the immune system needs to be heavily mobilized against [38]. However, this idea has been challenged by the observation that *Bacteroides* can produce an antagonistic form of lipopolysaccharide (LPS) in the gut that influences susceptibility to allergies and autoimmunity, which turns out to be a potential activator of innate immunity [39]. With the help of metagenomic sequencing analysis it was revealed that *Bacteroidetes* species contribute 79% of the LPS biosynthesis in healthy volunteers [40], thus driving immune silencing for the entire microbial community [40]. Hennezel et al. [40] also reported that the abundance of several *Bacteroidales* species (*Alistipes putredinis*, *Bacteroides caccae* and *Alistipes finegoldii*) show a moderate to strong correlation with the inhibition of IL-6 cytokine production, as well as TNF-α and IL-1β production. Moreover, it appears that the total LPS produced by the human gut microbiome not only is itself nonimmunogenic but also inhibits TLR4-dependent cytokine production [40]. It cannot be excluded that severe COVID-19 infection is associated with the activation of innate immune receptors, such as TLR-4. Indeed, it is well documented that severe COVID-19 infection is often associated with a cytokine storm [41], which is characterized by the overstimulation of macrophages, dendritic cells and monocytes, producing the pro-inflammatory interleukin 1 (IL1), interleukin 6 (IL6), interleukin 10 (IL10), tumor necrosis factor α (TNF-α) and tumor necrosis factor β (TNF-β) [42]. It is noteworthy that severe COVID-19 infection, which almost always is accompanied by the acute respiratory distress syndrome, is very distinguishable from mild infection of COVID-19 in terms of cytokine, C-reactive protein and ferritin levels, as well as in the lack of eosinopenia in mild cases [42]. In the present investigation, except for two subjects, mild symptoms were noted in all subjects who did not suffer from a cytokine storm. Therefore, it cannot be excluded that an increased level of *Bacteroidetes* could be part of the immune defense system by inhibiting TLR-4 activation. This hypothesis needs further investigation.

The other mechanisms by which *Bacteroidetes* could support the immune response against COVID-19 is by the downregulation of ACE2 receptors, which provide the main entrance of the virus into the cells, especially in the respiratory and gastro-intestinal tracts [43]. Indeed, *Bacteroidetes* have been demonstrated to suppress ACE2 levels [44]. During the second wave of this pandemic, when the original form of the COVID-19 virus was dominant in Europe, patients with enhanced age, with chronic inflammation, with obesity or with diabetes mellitus were the most sensitive populations for severe infection or an elevated ratio of mortality [45]. Interestingly, a comparison of the fecal microbiomes of these selected populations with those of healthy individuals demonstrates that the *Bacteroidetes* levels of old subjects, or patients with chronic inflammation, diabetes mellitus and/or obesity, are suppressed [46,47,48]. This again supports our hypothesis that increased levels of *Bacteroides* during mild levels of COVID-19 infection in young trained and sedentary subjects could have a protective immune system supporting effect.

The increased levels of Bacteroides vulgatus, which were observed during COVID-19 infection compared to samples collected three weeks following a negative PCR test, could suggest that the infection could result in subsequent infiltration of acute inflammatory cells through the gut barrier and result in the development of colitis symptoms [49].

In summary, contrary to our expectation, we could not detect significant differences in the fecal microbiome flora of trained and sedentary subjects, although it must be mentioned that almost all subjects reported mild symptoms of COVID-19. The major finding of the current study is that during mild COVID-19 infection the levels of *Bacteroidetes* are enhanced, which could support the immune system by suppressing the activation of TLR4 and ACE2 receptors. Further investigation is necessary and guaranteed.

## Figures and Tables

**Table 1:** Characteristics of the trained and control cohorts.

	Trained	Control
Gender, *n* (male/female)	16/4	15/5
Age, years	24.15 ± 4.75	27.75 ± 7.51
Height, cm	183.95 ± 10.38	175.5 ± 10.7
Weight, kg	83.93 ± 17.33	72.825 ± 12.75
Exercise hours per week	14.4 ± 6.2	1.25 ± 0.91
Smoker, *n*	3	2
Symptomatic group, *n*		
*Mild disease (2.)*	3	7
*Moderate disease (3.)*	15	13
*Severe disease (4.)*	2	0
*Critical disease (5.)*	0	0
Duration of illness, *n*		
*1–5 days (1)*	10	9
*5–14 days (2)*	10	10
*More than 14 days (3)*	0	1
Antibiotic treatment, *n*	1	2

Values are mean ± SD, or *n* as noted.

## Data Availability

The data presented in this study are available in article.
